# Identification of Oil-Loving *Cupriavidus necator* BM3-1 for Polyhydroxyalkanoate Production and Assessing Contribution of Exopolysaccharide for Vegetable Oil Utilization

**DOI:** 10.3390/polym16121639

**Published:** 2024-06-10

**Authors:** Yuni Shin, Hyun Joong Kim, Tae-Rim Choi, Suk Jin Oh, Suwon Kim, Yeda Lee, Suhye Choi, Jinok Oh, So Yeon Kim, Young Sik Lee, Young Heon Choi, Shashi Kant Bhatia, Yung-Hun Yang

**Affiliations:** 1 Department of Biological Engineering, College of Engineering, Konkuk University, Seoul 05029, Republic of Korea; sdbsdl0526@naver.com (Y.S.); sopero2@naver.com (H.J.K.); srim1004@gmail.com (T.-R.C.); equal73@naver.com (S.J.O.); rlatn990@naver.com (S.K.); qux1026@gmail.com (Y.L.); suhye0823@konkuk.ac.kr (S.C.); xmfvm@naver.com (J.O.); shashibiotechhpu@gmail.com (S.K.B.); 2 Innovation Center, Lotte Chemical Ltd., Seoul 07594, Republic of Korea; soyeon_kim1@lotte.net (S.Y.K.); lee.ys@lotte.net (Y.S.L.); knh508@lotte.net (Y.H.C.); 3 Institute for Ubiquitous Information Technology and Application, Konkuk University, Seoul 05029, Republic of Korea

**Keywords:** *Cupriavidus necator* BM3-1, oil, EPS, polyhydroxyalkanoates, P(3HB-*co*-3HHx)

## Abstract

Polyhydroxyalkanoates (PHA) have received attention owing to their biodegradability and biocompatibility, with studies exploring PHA-producing bacterial strains. As vegetable oil provides carbon and monomer precursors for poly(3-hydroxybutyrate-*co*-3-hydroxyhexanoate) (P(3HB-*co*-3HHx)), oil-utilizing strains may facilitate PHA production. Herein, *Cupriavidus necator* BM3-1, which produces 11.1 g/L of PHB with 5% vegetable oil, was selected among various novel *Cupriavidus necator* strains. This strain exhibited higher preference for vegetable oils over sugars, with soybean oil and tryptone determined to be optimal sources for PHA production. BM3-1 produced 33.9 g/L of exopolysaccharides (EPS), which was three-fold higher than the amount produced by H16 (10.1 g/L). EPS exhibited 59.7% of emulsification activity (EI24), higher than that of SDS and of EPS from H16 with soybean oil. To evaluate P(3HB-*co*-3HHx) production from soybean oil, BM3-1 was engineered with P(3HB-*co*-3HHx) biosynthetic genes (*phaC_Ra_*, *phaA_Re_*, and *phaJ_Pa_*). BM3-1/pPhaCJ produced 3.5 mol% of 3HHx and 37.1 g/L PHA. BM3-1/pCB81 (phaCAJ) produced 32.8 g/L PHA, including 5.9 mol% 3HHx. Physical and thermal analyses revealed that P(3HB-*co*-5.9 mol% 3HHx) was better than PHB. Collectively, we identified a novel strain with high vegetable oil utilization capacity for the production of EPS, with the option to engineer the strain for P(3HB-*co*-3HHx).

## 1. Introduction

Polyhydroxyalkanoates (PHAs) are organic biodegradable polymers produced by various microorganisms under unbalanced nutrient conditions, such as limited nitrogen or phosphorous and excess carbon [1]. PHA has been proposed as an alternative resource, replacing petroleum-based plastics, which negatively affect the environment [2,3]. PHAs can be classified into several types based on their composition. Further, they exhibit different properties and characteristics depending on the monomer composition [4]. For example, polyhydroxybutyrate (PHB) consists of only four carbon subunits of 3-hydroxybutyrate. PHB is relatively stiff and less flexible than petroleum-based plastics, which makes it difficult to process [5]. Copolymers, such as poly(3-hydroxybutyrate-co-3-hydroxyhexanoate) and P(3HB-*co*-3HHx), have properties similar to those of petroleum-based plastics, with high flexibility, low stiffness, and low crystallinity. Since copolymers are used in various industries owing to their properties, extensive research has been conducted to identify copolymer-producing bacterial strains [6,7].

*Cupriavidus necator*, also known as *Ralstonia eutropha*, is a PHA-producing strain capable of accumulating large amounts of PHAs, that is, up to 70–80% of its dry cell weight [8]. Wild-type *C. necator* can produce only PHB without additional precursors [9]. However, strains can be engineered to produce diverse copolymers, such as poly-3-hydroxybutyrate-*co*-3-hydroxyvalerate (P(3HB-*co*-3HV)), poly-3-hydroxybutyrate-*co*-4-hydroxybutyrate (P(3HB-*co*-4HB)), and poly-3-hydroxybutyrate-*co*-3-hydroxyhexanoate P(3HB-*co*-3HHx), using various substrates as the sole carbon source [10]. With regard to P(3HB-*co*-3HHx), there have been many reports on *Cupriavidus* strains engineered through the insertion of PHA synthase (*phaC*) derived from *Aeromonas caviae*, *Rhodococcus aetherivorans*, mangrove soil (PhaCBP-M-CPF4), *Chromobacterium* sp. USM2 and *Pseudomonas* sp. 61–3, and enoyl-CoA hydratase (*phaJ*) derived from *Pseudomonas aeruginosa* and *Streptomyces* sp. CFMR7 for poly(3-hydroxybutyrate-*co*-3-hydroxyhexanoate) production. These engineered strains could efficiently produce P(3HB-*co*-3HHx) using short-chain fatty acids or plant oils as precursors [11]. 

Plant oils are suitable precursors for PHA production, as they provide more carbon units than sugars and are located downstream of sugars in the PHA production route, making them easier to utilize [12,13]. In addition, plant oils can provide precursors of 3-3HHx via *phaJ*, which is an effective carbon source for P(3HB-*co*-3HHx) production. Therefore, using strains that efficiently utilize oil can help increase PHA production [14,15], which, when coupled with genetic tools, can give rise to potent P(3HB-*co*-3HHx)-producing strains. Considering that most studies have used *Cupriavidus necator* H16-based engineered strains, identifying new strains may be helpful for the study of P(3HB-*co*-3HHx). 

Exopolysaccharide (EPS) is a high-molecular-weight polymer produced by microorganisms. It consists of carbohydrates, DNA, proteins, and phospholipids, as well as non-carbohydrates, such as acetate, glycerol, and phosphates. EPS is secreted into the extracellular medium or attached to the cell surface. Microorganisms produce EPS for several reasons. For example, they serve as a carbon or energy source under nutrient shortage conditions [16,17,18,19]. Owing to their structural characteristics, EPS has various functional properties, including emulsification properties and flocculating activity, as well as antimicrobial and antioxidant activities [19]. These characteristics render EPS widely applicable in various industries, including pharmaceuticals, wastewater treatment, and cosmetics [20]. 

While numerous *C. necator* strains are recognized for EPS production, a limited number of studies have explored the relationship between oil uptake and EPS synthesis. To address this knowledge gap, in this study, we aimed to identify novel *C. necator* strains with enhanced PHA production capabilities. After selecting an oil-loving *Cupriavidus*, the major nutrient sources, including carbon and nitrogen, were optimized. Additionally, we investigated the role of exopolysaccharides (EPS) to elucidate the efficient utilization of oil by this strain. Furthermore, we engineered a strain to exclusively utilize oil as a carbon source for cell growth, maintenance, and P(3HB-*co*-3HHx) production. We believe that our findings may highlight the potential for cost-effective co-production of EPS and P(3HB-*co*-3HHx) in industrial applications.

## 2. Materials and Methods

### 2.1. Strains, Plasmids, and Identification

All strains and plasmids used in this study are listed in Table 1. Nine strains, excluding *Cupriavidus necator* H16, were obtained from the Korea Environmental Microorganisms Bank (KEMB; Suwon, Republic of Korea). Each stock sample was suspended with distilled water and spread on Tryptic Soy Agar (TSA) with 10 µg/L gentamicin for isolation. The plate was incubated for 48 h at 30 °C. Isolated single colonies from each plate were cultured for 24 h in Tryptic Soy Broth (TSB; MB cell, Seoul, Republic of Korea) with 10 µg/L gentamicin (*Ralstonia eutropha* exhibits a gentamicin resistance) at 30 °C, and at 200 rpm. Stocks were prepared with 20% (*v*/*v*) glycerol and stored at −81 °C.

The strain was identified at the species level via 16S rRNA sequencing. Partial 16s rRNA was acquired from Bionics (Seoul, Republic of Korea) via polymerase chain reaction amplification using primers 27F and 1492R. This sequence was compared to the NCBI GenBank database via BLASTN tools and used to construct a phylogenetic tree using Molecular Evolutionary Genetics Analysis version 11 (MEGA 11) (Tamura, Stecher, and Kumar 2021).

### 2.2. Culture Conditions and Medium Optimization

For the identification of PHA-producing strains and medium optimization, a seeding medium was prepared by culturing the strain at 30 °C for 24 h in 5 mL of TSB with 10 µg/L gentamicin. The cells were then harvested and washed twice with sterilized water. Subsequently, a 2% (*v*/*v*) seed culture was used to inoculate 5 mL of cell culture media. The cell culture medium was based on *Ralstonia eutropha* minimal medium (ReMM), with an initial pH of 6.8. The composition included 8 g/L NaH_2_PO_4_, 9.2 g/L Na_2_HPO_4_, 0.45 g/L K_2_SO_4_, 0.39 g/L MgSO_4_, 62 mg/L CaCl_2_, and 1 mL per liter of trace element solution. The trace element solution consisted of 15 g/L FeSO_4_⋅7H_2_O, 2.4 g/L MnSO_4_⋅H_2_O, 2.4 g/L ZnSO_4_⋅7H_2_O, and 0.48 g/L CuSO_4_⋅5H_2_O dissolved in 0.1 M hydrochloric acid [23]. During the experiments, carbon and nitrogen sources were varied. For screening, 0.2% (*w*/*v*) urea served as the nitrogen source, whereas 5% (*v*/*v*) soybean oil and 1% (*w*/*v*) fructose were used as carbon sources. Carbon source optimization was conducted using a formulation of 1% differing carbon sources and 0.2% (*w*/*v*) urea. Nitrogen source utilization was explored using various organic and inorganic nitrogen sources, with 5% oil as the carbon source. Unless specified otherwise, medium components were procured from Sigma-Aldrich (St. Louis, MO, USA).

### 2.3. Crude EPS Characterization

#### 2.3.1. Crude EPS Production and Extraction

**Figure 1:**
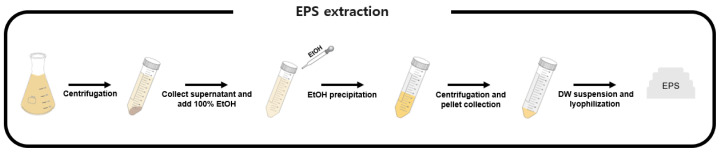
Crude EPS extraction method. Simple scheme of crude EPS extraction method.

For EPS production, 100 mL working volume in a 250 mL flask culture was used. Strain BM3-1 was used for production, and *C. necator* H16 was used as a control. ReMM was used for culture medium, with 5% (*v*/*v*) soybean oil and 1% (*w*/*v*) fructose as carbon sources, and strains were cultured at 30 °C. After 72 h incubation (Figure 1), cell-free supernatant was obtained via centrifugation at 3700 rpm for 30 min at 25 °C. The collected supernatant was precipitated in 100% absolute cold ethanol with a three-fold volume and incubated at 4 °C for 24 h. The precipitate was collected via centrifugation at 3700 rpm for 15 min at 4 °C. Only the pellets were used for further experiments. The EPS pellet was resuspended in distilled water and lyophilized. After lyophilization, crude EPS was extracted and stored at room temperature until further use.

#### 2.3.2. Gas Chromatography–Mass Spectrometry (GC-MS) for Analysis of EPS Composition

The monosaccharide composition of crude EPS was analyzed using GC-MS. Before analysis, 20 mg of dried EPS was hydrolyzed with 2 M trifluoroacetic acid at 110 °C for 3 h. The hydrolysate was dried and washed thrice with 500 μL of methanol. The residue was dissolved in pyridine, and the derivatization of samples was completed using N-methyl-N-(trimethylsilyl) trifluoroacetamide (MSTFA), followed by incubation at 70 °C for 10 min. This solution was filtered into GC vials using a 0.22-μm PVDF filter. GC-MS analysis (Calrus 500; Perkin Elmer, Waltham, MA, USA) was used to determine the monosaccharide composition of the prepared samples. One microliter of sample was injected into an Elite 5 ms column (30 m × 0.25 mm × 0.25 μm film thickness) with helium as the carrier gas at a split ratio of 10:1 and a column flow of 1.0 mL/min. The oven and column temperatures were programmed as 60 °C for 2 min, then increased to 140 °C at a rate of 2 °C/min, held for a second, then again increased to 300 °C at 20 °C/min and held at that temperature for 10 min. The temperatures of the injector and ion sources were set to 280 and 250, respectively. The electron energy was set to 70 eV, and the entire scan range was 45–400 m/z, with a scan time of 0.2 s. The derivatized compounds were identified using the NIST library [24].

#### 2.3.3. Crude EPS Production and Extraction

To assess the emulsifying activity of 1% (*w*/*v*) crude EPS, five hydrocarbons were used, namely, hexadecane, methyloctanoate, methyl 10-undecenoate, toluene, and soybean oil. These hydrocarbons were added to 1% EPS at a ratio of 1:1. After vortexing for 2 min, the mixture was incubated overnight at room temperature. The emulsification index was then calculated as follows [25]:

$$Emulsification\;index\;(EI24,\%)=\frac{Height\;of\;the\;emulsified\;layer}{Height\;of\;the\;total\;solution}\times 100\%$$

For the control, 1% SDS was used in the same manner as the five hydrocarbons.

#### 2.3.4. Flocculation Activity

The flocculating activity was assessed using a solution of natural kaolinite (kaolin clay) (Al_2_Si_2_O_5_(OH)_4_) and 0.5% (*w*/*v*) of crude EPS. A mixture of 4 mL of 1% (*w*/*v*) CaCl_2_ and 200 μL of crude EPS solution was combined with a 50 mL solution of 0.5% (*w*/*v*) kaolin clay at pH 8.0. The mixture was agitated gently for 2 min and allowed to settle for 5 min at room temperature. The clear aqueous phase was separated, and its optical density (OD) was measured at 550 nm using a 96-well plate read on a UV/visible spectrophotometer (Biotek, Winooski, VT, USA). The flocculation efficacy was determined using a specific formula [26]:

$$Flocculating\;activity\;(\%)=\frac{A-B}{A}\times 100\%$$

where A and B are the optical densities at 550 nm for distilled water (control) and EPS samples, respectively. The effects of the pH of the solution on flocculating activity were also examined. The pH of the kaolinite suspension was adjusted using 1 M NaOH and 1 M HCl in the pH range of 3–9.

### 2.4. Vector Construction and Transformation

For P(3HB-*co*-3HHx) production, plasmids pCB81 and pPhaCJ were introduced into BM3-1 cells via electroporation. pPhaCJ plasmid harbors *phaC* from *Rhodococcus aetherivorans* and *phaJ* from *Pseudomonas aeruginosa*. These sequences were codon-optimized for *Ralstonia eutropha*, so the process was synthesized by gene synthesis. These genes were placed between the KpnI and the HindIII restriction enzyme in the MCS site. The strain was cultured in 5 mL TSB with 10 µg/L gentamicin-containing medium for 24 h and centrifuged. Cell pellets were washed thrice and resuspended in 10% chilled glycerol. The prepared plasmid and suspended cells were added to a 0.2-cm cuvette and electroporated using a MicroPulser (Bio-Rad, Hercules, CA, USA). Electroporated cells were recovered in 1 mL of TSB medium for 2 h and selected in TSA with 10 µg/L gentamicin and 300 µg/L kanamycin for reliable selection. The selected colonies were incubated in TSB with 10 µg/L gentamicin and 100 µg/L kanamycin. Subsequently, stocks were stored with 20% (*v*/*v*) glycerol at −81 °C for further experiments.

### 2.5. Fed-Batch Fermentation

Fermentation was performed to produce P(3HB-*co*-3HHx). Before fermentation, seed cultures were prepared, and engineered BM3-1 strains were precultured in 5 mL TSB medium with 10 µg/L gentamicin and 100 µg/L kanamycin, then incubated for 24 h at 30 °C, at 200 rpm. Thereafter, the seed culture was transferred to 250 mL baffled flasks containing 100 mL ReMM (explained above), 1% fructose, 0.2% (*w*/*v*) urea, and trace element, as well as 10 µg/L gentamicin and 100 µg/L kanamycin, cultured for 24 h at 30 °C, at 200 rpm. 

Fed-batch fermentation was performed using a 2 L working volume in a 5 L fermenter (CNS; Daejeon, Republic of Korea). The ReMM-based medium described earlier was used for fermentation. For carbon sources, 1% fructose (*w*/*v*) and 5% oil (*v*/*v*) were used. Approximately 0.2% (*w*/*v*) urea was used as a nitrogen source. Twenty milliliters of the inoculum were added and the remainder filled with distilled water, to a final volume of 2 L. The pH was adjusted to pH 6.5 using NaOH and HCl. After 18 h of fermentation, additional oil was fed into batch fermentation at a feeding rate of 6.9 g/L. Additional feeding was stopped after 300 g of oil had been supplied. During fermentation, intermediate sampling was conducted to determine cell growth and PHA production. Cells harboring pPhaCJ and pCB81 were harvested at 133 and 115 h, respectively. The harvested cells were used to prepare the PHA film and calculate the dry cell weight (DCW), PHA, and 3HHx mole fractions.

### 2.6. PHA Analysis

#### 2.6.1. Gas Chromatography—Flame Ionization Detector (GC-FID)

After incubation, 1 mL of the culture medium was used for DCW and PHA production. One milliliter of the medium was centrifuged and washed once with distilled water, then washed twice with hexane to remove soybean oil, which was followed by lyophilization. Using the lyophilized cells, the DCW was calculated as the difference between the original glass vial weight and the glass vial with the lyophilized cell. For GC-MS analysis, the fatty acid methyl ester (FAME) method was used for derivatization. One milliliter of chloroform and 1 mL 15% (*v*/*v*) H_2_SO_4_/85% (*v*/*v*) methanol solution was added to the lyophilized cell pellet and incubated at 100 °C for 2 h. After cooling down the methyl ester solution, 1 mL of distilled water was added and vortexed briefly. Only the chloroform layer was used, and trace amounts of water were removed using sodium anhydride. Samples were filtered to GC vials using a 0.22-μm PVDF filter. One-microliter aliquots of the sample were introduced into a gas chromatograph operating in split mode (1/10) (Young-lin 6500, Seoul, Republic of Korea). The chromatograph was equipped with a fused silica capillary column (Agilent HP-FFAP, 30 m × 0.32 mm, i.d. 0.25 μm film) and an FID. Helium served as the carrier gas, flowing at a rate of 3 mL/min, and the inlet temperature was set to 210 °C. The oven temperature followed a gradient program, 0–5 min at 80 °C, and followed by 12–17 min at 220 °C, while maintaining the FID temperature at 230 °C throughout the entire experiment. PHB was quantified using a previously established regression equation calibrated with authentic P(3HB-*co*-3HHx) as a reference. PHB content was determined by computing the percentage of PHB produced relative to the total DCW. For external standards, poly(3-hydroxybutyratic acid) and poly(3-hydroxybutyrate-co-3-hydroxyhexanoate) were used. For the internal standard, benzoic acid was used.

#### 2.6.2. Film Production

For film production, engineered strains were cultivated from 5 L of fermentation, whereas the wild-type strain was obtained from a 100 mL flask culture using a 250 mL flask. The cell pellet was collected via centrifugation, washed once with distilled water, washed twice with hexane, and lyophilized. Subsequently, 25 mL of chloroform was added to the lyophilized cells for cell lysis and chloroform extraction was conducted at 60 °C for 2 h. The chloroform layer containing the dissolved polymer was separated from the cellular debris via centrifugation and filtered using Whatman No. 1 filter paper to eliminate any remaining cellular residues. The resulting PHB solution was poured into a glass Petri dish, and a PHA film was formed after the chloroform was evaporated at room temperature. The thermal and physical characteristics of the film were assessed using gel permeation chromatography (GPC), a universal testing machine (UTM), and differential scanning calorimetry (DSC), respectively.

#### 2.6.3. GPC

A GPC instrument (YL Chromass; Anyang, Republic of Korea) featuring a loop injector (Rheodyne 7725i) (Thermo Scientific; Waltham, MA, USA), an isocratic pump with dual heads (YL9112) (YL Chromass; Anyang, Republic of Korea), a column oven (YL9131) (YL Chromass, Anyang, Republic of Korea), a column (Shodex K-805, 8.0 I. D. × 300 mm) (Shodex; Tokyo, Japan), and an RI detector (YL9170) (YL Chromass; Anyang, Republic of Korea) was used for the GPC analysis. A solution was prepared by dissolving 10 mg of the PHB film in 2 mL of chloroform. The solution was then filtered using a 0.2-μm PVDF filter. An air bubble-free solution of 60 μL was injected into the GPC instrument. Chloroform served as the mobile phase at a flow rate of 1 mL/min, with a constant temperature of 35 °C. Molecular masses were compared to polystyrene standards (ranging from 5000–2,000,000 Da) using the YL-Clarity software (ver. 8.3) specifically designed for a single YL HPLC instrument.

#### 2.6.4. UTM

The mechanical properties of the BM3-1-derived PHA film were analyzed using an EZ-SX universal testing machine (UTM; Shimadzu, Kyoto, Japan). PHA films were prepared in a rectangular shape, with dimensions of 60 mm × 10 mm (length × width). The thickness was measured using a 1111–100 A mini digital caliper (Insize, Loganville, GA, USA). The analysis conditions were set to a rate of 20 mm/min. The tensile strength, elongation at break, and Young’s modulus were calculated from graphs plotted using TRAPEZIUM X software (ver 1.5.5).

#### 2.6.5. DSC

To determine the thermal properties of the BM3-1-derived film, DSC analysis was conducted using a NEXTA DSC 200 instrument (Hitachi, Tokyo, Japan) to calculate Tg, Tc, and Tm. The analysis was performed within a temperature range of −60 °C to 180 °C, with a heating and cooling rate of 10 °C/min in an N_2_ atmosphere.

## 3. Results and Discussion

### 3.1. Screening PHA-Producing C. necator Strains Obtained from KEMB

**Figure 2:**
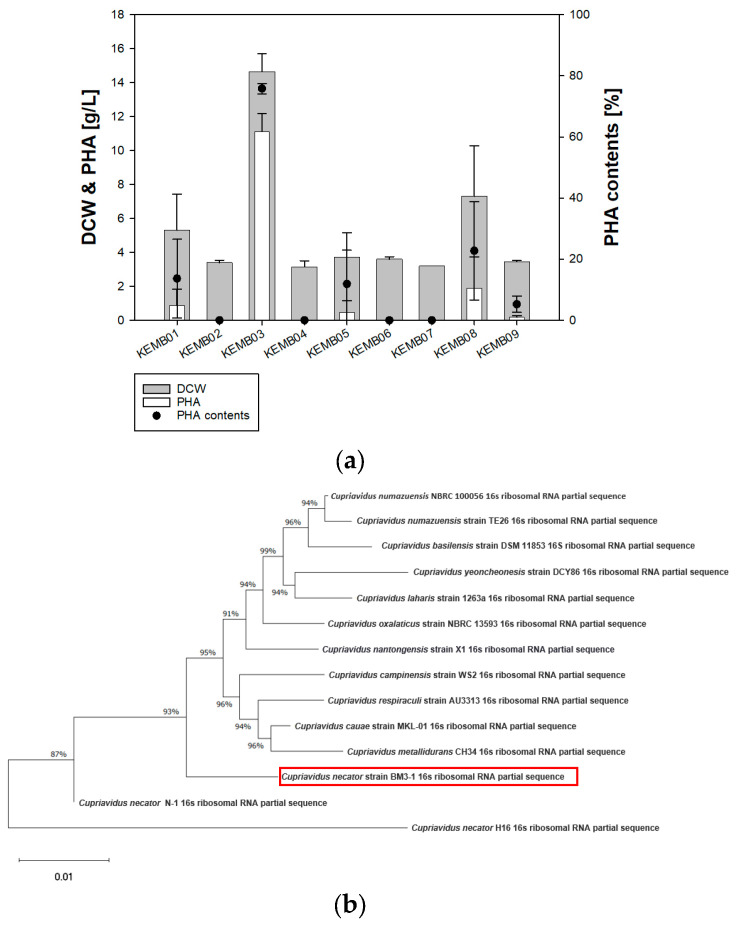
Screening and identification of PHA-producing *Cupriavidus necator* strains. (**a**) Comparison of DCW and PHA production by nine *Cupriavidus necator* strains via GC analysis. Strains were cultured in *Ralstonia* minimal medium, with 1% fructose and 5% soybean oil. (**b**) Phylogenetic tree generated via 16S rRNA sequencing of *Cupriavidus necator* BM3-1.

As there has been no research on these nine strains from KEMB, PHB production and growth were evaluated. Usually, *C. necator* uses fructose and plant oils as the sole carbon sources in its synthesis of PHB. Therefore, we added fructose and soybean oil to *Ralstonia* minimal medium to screen PHA-producing strains. As a result, all strains were cultured in *Ralstonia* minimal medium, with 1% fructose and 5% soybean oil (Figure 2a). Among them, only five PHA-accumulating strains were confirmed using GC analysis. Among them, *C. necator* KEMB03, in the same manner as *C. necator* BM3-1, produced the highest amount of PHA (14.6 g/L of DCW, 11.1 g/L of PHA) (Table 1, Figure 2). Therefore, *C. necator* BM3-1 was selected for further studies on PHA accumulation. *C. necator* BM3-1, used for 16s rRNA analysis, demonstrated the highest similarity with *C. necator* N-1 through phylogenetic analysis (Figure 2b).

### 3.2. Optimization of Carbon and Nitrogen Sources for Growth

**Figure 3:**
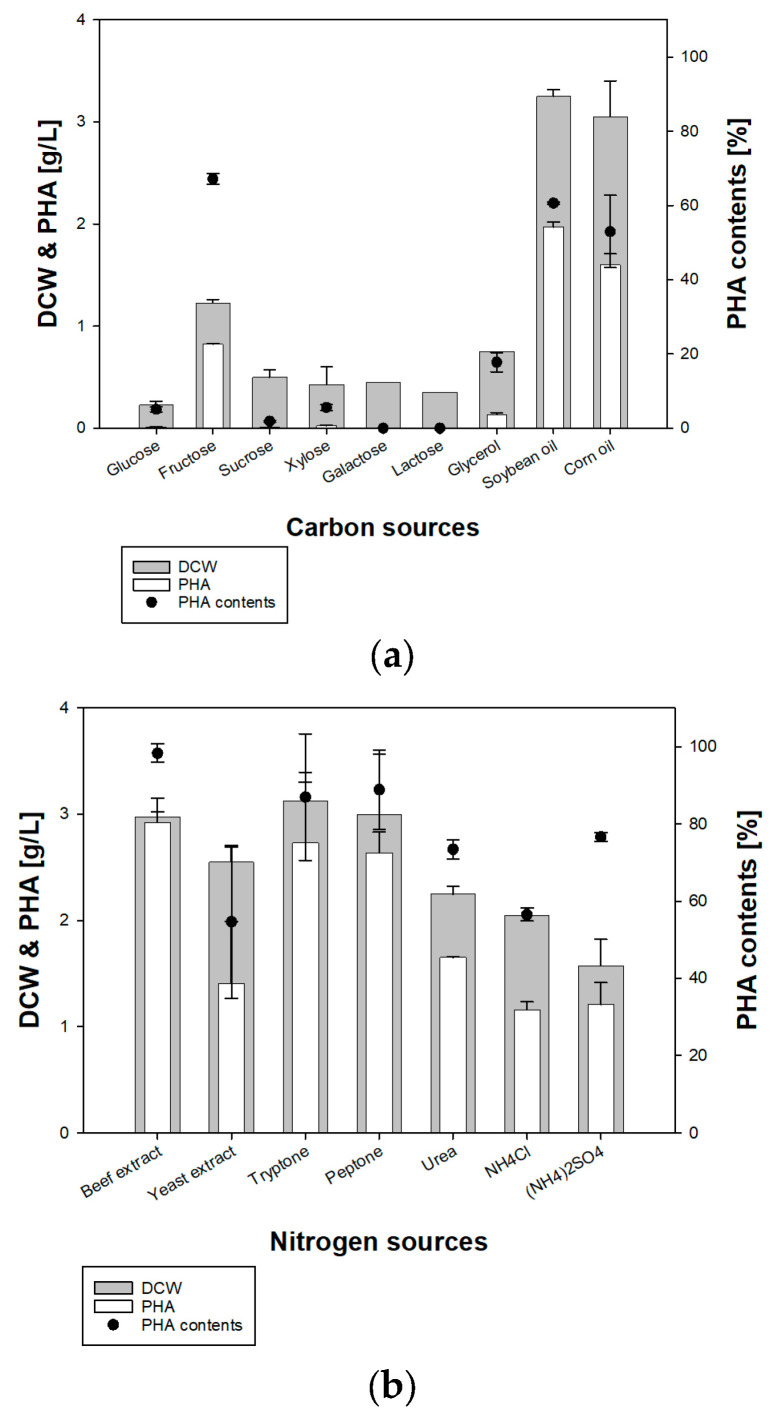
Optimal culture conditions for cell growth and PHA production. (**a**) Effects on cell growth and PHA production in carbon sources ranging from sugars to oils (glucose, fructose, sucrose, xylose, galactose, lactose, glycerol, soybean oil, and corn oil). (**b**) Comparison of growth and PHA production with different types of inorganic nitrogen sources (urea, NH_4_Cl, and (NH_4_)_2_SO_4_) and organic (beef extract, yeast extract, tryptone, and peptone) nitrogen sources.

To determine optimal growth conditions for the BM3-1 strain, we identified the carbon and nitrogen sources that had the most positive impact on growth and PHA accumulation (Figure 3). The concentration of carbon sources was fixed at 1%, whereas that of nitrogen sources was fixed at 0.2% of the final volume. For carbon source optimization, 0.2% (*w*/*v*) of urea was used, and 5% soybean oil was used for nitrogen source optimization.

For carbon sources, a range of sugars and oils (glucose, fructose, sucrose, xylose, galactose, lactose, glycerol, soybean oil, and corn oil) were used. Strain BM3-1 grew well when using oil-based sources, such as soybean oil and corn oil. Soybean oil was the best carbon source, and fructose facilitated the best growth among the sugars. Strain BM3-1 showed the highest growth and PHA production when soybean oil was used, at 3.25 g/L DCW and 1.95 g/L, respectively. This pattern was similar to that of *C. nectator* H16, which resulted in 3.5 g/L of DCW with 1.4 g/L of PHA from fructose [27] and about 6 g/L of DCW, with 80% of PHA contents from oil, although it used different conditions in detail [22,28].

As for nitrogen sources (beef extract, yeast extract, tryptone, peptone, urea, NH_4_Cl, and (NH_4_)_2_SO_4_), organic and inorganic nitrogen sources were confirmed. Most organic nitrogen sources, such as beef extract, tryptone, and peptone, resulted in among the best growth levels. Among these complex N sources, tryptone facilitated the highest growth and PHA production at 3.12 g/L DCW, 2.72 g/L PHA, and 86.5% PHA contents. The complex nitrogen source was optimal, but owing to economic efficiency and exact results, urea, which showed the best cell growth and PHA production among inorganic N sources (2.25 g/L DCW, 1.65 g/L PHA, and 73.3% PHA contents), was used for further research.

### 3.3. Extraction and Analysis of Crude EPS

During cell recovery, sedimentation and centrifugation at low rpm were difficult, unlike for *C. necator* H16. To determine which affected sedimentation in the supernatant, we evaluated the materials in the supernatant. By applying the EPS sedimentation protocol described in the Materials and Methods section, a large amount of EPS in the supernatant was detected when *C. necator* H16 was used as the control for EPS production. Compared with *C. necator* H16, BM3-1 produced 33.9 g/L of crude EPS, which was approximately three-fold higher than the amount produced by control strain H16 (10.1 g/L).

**Figure 4:**
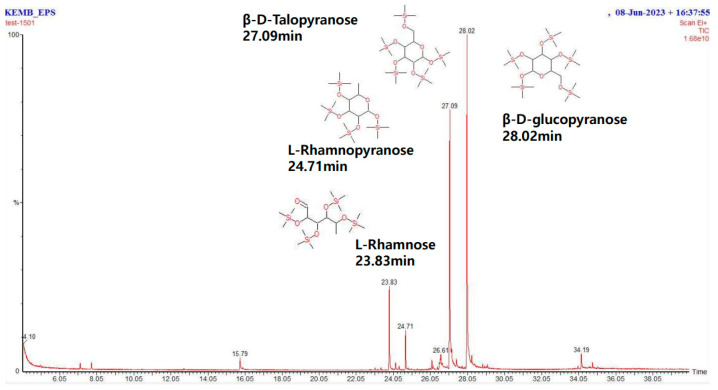
EPS composition analysis using GC-MS. Monosaccharide analysis using GC-MS through MSTFA derivatization.

To determine the specific characteristics of crude EPS, various characterizations were performed. The composition of the crude EPS was analyzed using GC-MS. The major peak of the GC-MS chromatogram (Figure 4) was further characterized. It was mainly composed of L-Rhamnose, L-Rhamnopyranose, β-D-Talopyranose, and β-D-glucopyranose, with the latter three being the major components. The proportion of each molecule was calculated by a semi-quantitative method based on the peak areas of the four molecules (Table 2). β-D-glucopyranose was the major component of crude EPS from BM3-1, at about 46%. 

**Figure 5:**
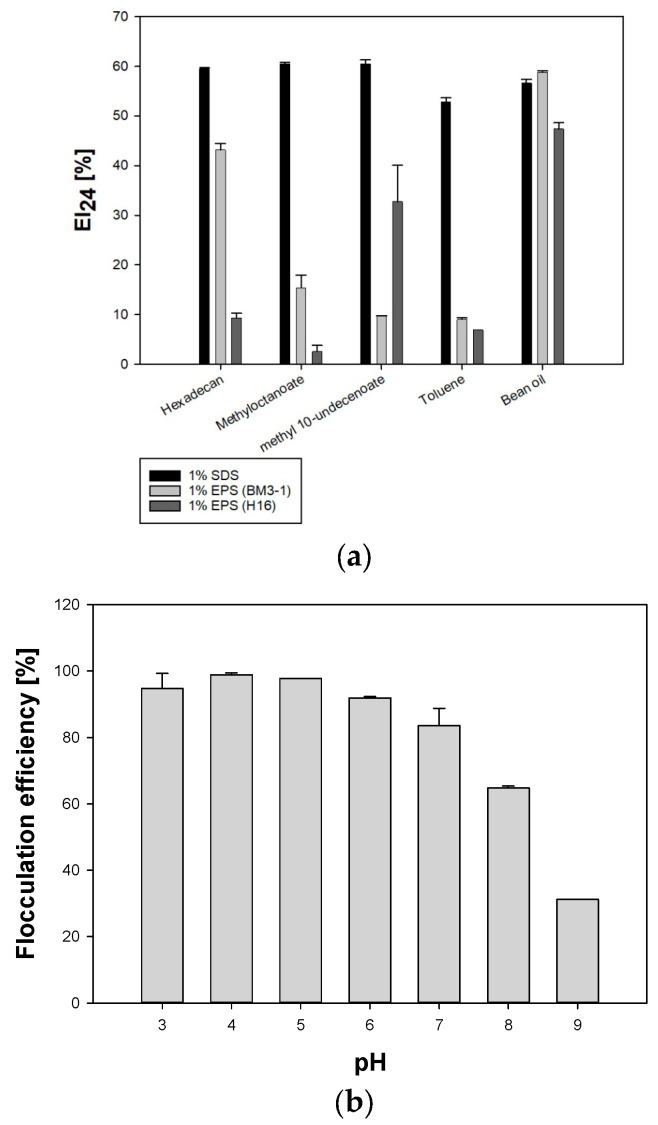
Characterization of crude EPS properties of BM3-1. (**a**) Emulsification activity of EPS. The 1% SDS and 1% EPS from H16 were used as controls. (**b**) Comparison of flocculation efficiency according to pH (pH 3 to 9).

Subsequently, the emulsification activity of crude EPS from BM3-1 was tested (Figure 5a). SDS, a chemical emulsifier, was used as the control surfactant. EPS produced from H16 was also used as a control for comparing emulsifying activity. SDS (1%) exhibited higher emulsifying activity than 1% BM3-1 EPS in hexadecane, methyloctanoate, methyl 10-undecenoate, and toluene, with emulsification indices of 59.5%, 60.5%, 60.4%, and 52.8%, respectively. However, the crude EPS exhibited the maximum emulsification activity in soybean oil, at 59.7%, which was higher than that of 1% SDS (56.5%). When comparing 1% BM3-1 EPS with 1% H16 EPS, those from BM3-1 showed higher emulsification activity, except for methyl 10-undecenoate. This difference was particularly pronounced in soybean oil; BM3-1 (58.7%) had higher EI24 than H16 (47.4%). This result demonstrates that the EPS produced by strain BM3-1 facilitates adequate mixing of media components and substrate access during growth. 

As flocculation activity helps metal ion binding and is affected by pH, we measured the flocculating activity in response to pH changes. The results (Figure 5b) suggest that the flocculation activity slightly increased as the pH increased from 3 to 4. After reaching a maximum (98.7%) at pH 5, flocculation activity was maintained until pH 6, then decreased at higher pH levels. After passing pH 8, the flocculating activity rapidly decreased to 31.1% at pH 9. As BM3-1 was cultured within the pH range of 6–7, flocculating activity was maintained during growth without inhibiting substrate uptake.

### 3.4. Fed-Batch Fermentation for P(3HB-co-3HHx) Production Using Engineered BM3-1

**Figure 6:**
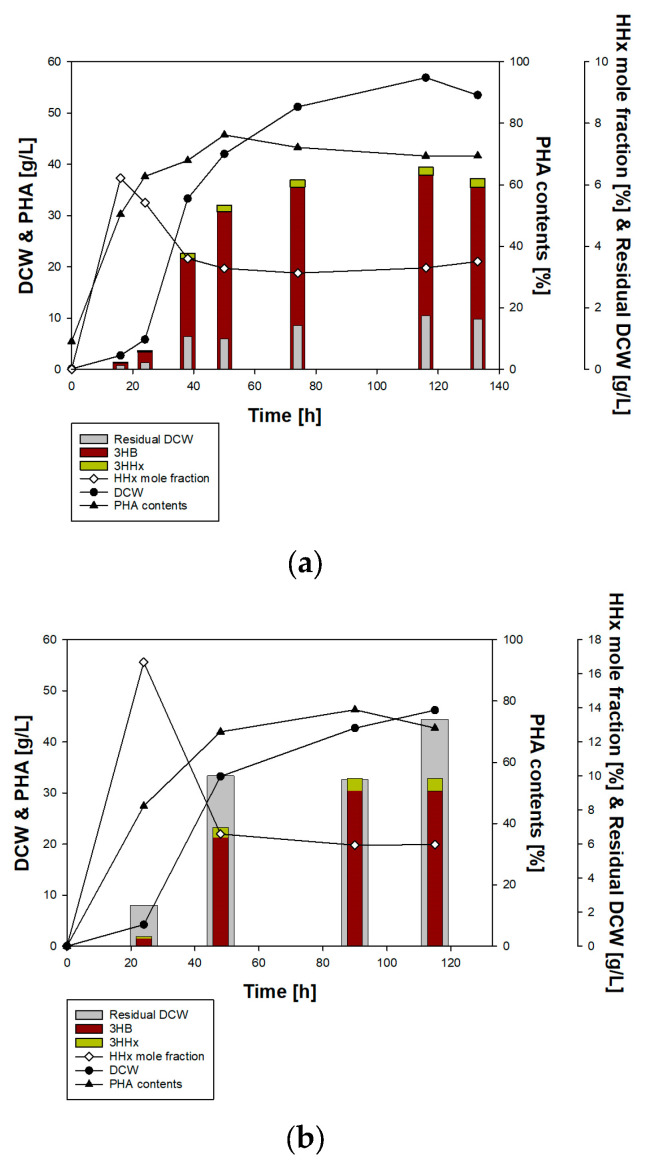
Fed-batch fermentation for P(3HB-co-3HHx) production using (**a**) BM3-1/pPhaCJ and (**b**) BM3-1/pCB81.

To produce P(3HB-*co*-3HHx), BM3-1 was engineered using a pCB81vector containing *phaC*, *phaA*, and *phaC*, as well as pPhaCJ containing *phaC* and *phaJ*, respectively. Before fermentation, 3HHx production was confirmed through a 5 mL culture using a 14 mL round tube. Subsequently, batch fermentation was conducted with 5% (*v*/*v*) soybean oil and 1% (*w*/*v*) fructose as carbon sources (Figure 6). Batch fermentation with BM3-1/pCB81 resulted in the highest PHA production, at 116 h, 39.4 g/L. At the end of fermentation, DCW was 53.5 g/L, the 3HHx mole fraction was 3.5 mol%, and this produced 37.1 g/L PHA. pPhaCJ showed the highest production of PHA (32.9 g/L, including 5.92 mol% of 3HHx). At the final point of fermentation, 32.82 g/L of PHA and 46.2 g/L of DCW were produced. The process yields of BM3-1/pCB81 and BM3-1/pPhaCJ were 0.18 gPHA/gCarbon source, and 0.16 gPHA/gCarbon source, respectively. BM3-1/pCB81 showed the higher process yield. However, in both strains, a gradual decrease in DCW and PHA was observed after 116 and 90 h, respectively. This was attributed to nutrient depletion. Furthermore, BM3-1 cells harboring pPhaCJ produced higher amounts of PHA, whereas those harboring pCB81 produced PHA with a higher 3HHx mole fraction. From the perspective of productivity of the fermentation, BM3-1/pPhaCJ showed higher productivity (0.28 g/L/h) than BM3-1/pCB81 (0.27 g/L/h).

### 3.5. Physical and Thermal Properties of PHA

After batch fermentation, the culture medium was harvested, and only the cell pellets were used for PHA film preparation. A BM3-1 wild-type cell pellet was produced using a 250 mL flask culture with a 100 mL working volume as the control. GPC and UTM analyses were performed to examine the physical properties of PHA produced by BM3-1. All samples were measured once.

Next, we sought to determine the average molecular weight (Mw), number average molecular weight (Mn), and polydispersity index (PDI = Mw/Mn) of the PHA films derived from BM3-1-based strains. Some differences existed between the wild-type and engineered strains (Table 3). Wild-type BM3-1 only produced PHB; this had an Mn of 8.85 × 10^5^, an Mw of 1.19 × 10^6^, and a PDI of 1.34. The Mn, Mw, and PDI values of BM3-1/pCB81 were 3.67 × 10^5^, 5.88 × 10^5^, and 1.60, respectively. The respective values for BM3-1/pPhaCJ were 3.65 × 10^5^ for Mn, 5.64 × 10^5^ for Mw, and 1.54 for PDI. As the engineered strains had 3HHx monomers, whereas the wild-type did not, their PDI values were higher. Furthermore, BM3-1/pCB81 had a higher 3HHx mole fraction than BM3-1/pPhaCJ, with higher Mn, Mw, and PDI values than those of BM3-1. 

The tensile strength, elongation at break, and Young’s modulus were evaluated using a universal testing machine (UTM) (Table 4). The PHB film produced from BM3-1 showed tensile strength, elongation at break, and Young’s modulus of 7.83 MPa, 24.8%, and 407.8 MPa, respectively. As 3HHx mol% increased, the film produced by BM3-1 pCB81 exhibited the lowest tensile strength and Young’s modulus at 5.22 MPa and 86.2 MPa, respectively. However, it showed the best elongation at break, 99.1%. In addition, BM3-1/pPhaCJ film exhibited superior physical properties, with a higher elongation at break than the BM3-1 PHB film. As mentioned in a previous study, an increase in the 3HHx mol% leads to an increase in the elongation at break and a decrease in the tensile strength and Young’s modulus [29]. Consistent results were also observed in the current study. 

Finally, the thermal properties of PHA films were determined via DSC (Table 5). The Tm and Tc of PHA film from BM3-1 were 174.3 °C and 96.0 °C, respectively, whereas the Tg was undetected. P(96.5 mol% 3HB-*co*-3.5 mol% 3HHx) film had a lower Tm than PHB film, at 170.2 °C. BM3-1 pPhaCJ film, having 5.9 mol% 3HHx, showed the lowest Tm at 162.3 °C, whereas the Tc was not calculated. This result was attributed to imperfect crystallization caused by a high 3HHx mol%. Consequently, having 3HHx monomers in PHA copolymers decreased the crystallinity, leading to improved mechanical properties. When the engineering *Ralstonia* strain was compared with other studies comparing the physical properties of PHBHHx made by culturing oil as a carbon source, the Tm value was 168.6 °C [30] and 151 °C [31], and the elongation at break was 132.2 ± 17.3% [28]. Through this, it was confirmed that BM3-1 also produces a similar form of PHBHHx, like other engineering *Ralstonia* strains. Based on low PDI values and one peak of Tg, this polymer seemed to be a random copolymer; however, considering the fact that both native PhaC and PhaC from *R. aetherivorans* could produce P(3HB-*co*-3HHx) with *phaJ*, there is a possibility of a blend of both P(3HB-*co*-3HHx) [29,32,33,34].

## 4. Conclusions

In this study, oil-utilizing *Cupriavidus necator* BM3-1 was observed to produce significant amounts of PHA (11.1 g/L of PHA). To determine optimal growth conditions, carbon and nitrogen sources were optimized, which are crucial for cell growth and PHA accumulation. BM3-1 demonstrated proficient utilization of oil and complex nitrogen sources. To identify the reason for high oil consumption, EPS production, which helps oil adsorption and cell growth, was confirmed. While wild-type *Cupriavidus necator* produced only PHB, limiting its industrial application, engineered strains were generated to produce copolymer film, such as P(3HB-*co*-3HHx). Two-liter fermentation using BM3-1 harboring pCB81 (39.4 g/L PHA) and pPhaCJ (37.1 g/L PHA) produced 5.9 and 3.5 mol% of 3HHx, respectively. PHA films obtained from fermentation were characterized via GPC, UTM, and DSC analyses which showed that PHA films from engineered films had improved physical properties compared to the PHA films from the wild-type. Through this study, the role and function of EPS in *Ralstonia eutropha* strains has been presented, and based on this result, P(3HB-*co*-3HHx) was produced through an engineered *Ralstonia eutropha* strain, with high EPS production.

## Figures and Tables

**Table 1:** Strains and plasmids used in this study.

Strain or Plasmid	Description	Simple Abbreviation	Reference or Source
*Cupriavidus necator* strains			
AM3-5	Wild-type strain, Gm resistance	KEMB01	KEMB 224-180
AM3-6	Wild-type strain, Gm resistance	KEMB02	KEMB 224-181
BM3-1	Wild-type strain, Gm resistance	KEMB03	KEMB 224-182
M19	Wild-type strain, Gm resistance	KEMB04	KEMB 224-233
M25	Wild-type strain, Gm resistance	KEMB05	KEMB 224-239
M93	Wild-type strain, Gm resistance	KEMB06	KEMB 224-307
M101	Wild-type strain, Gm resistance	KEMB07	KEMB 224-315
HK 32-1	Wild-type strain, Gm resistance	KEMB08	KEMB 1602-360
Cd9	Wild-type strain, Gm resistance	KEMB09	KEMB 2225-030
H16	Wild-type strain, Gm resistance		ATCC 17699
Plasmids			
pBBR1MCS-2	Vector for plasmid-based gene expression in *R. eutropha*, confers Km resistance		[21]
pCB81	pBBR1MCS-2-based plasmid with *phaC_Ra_* from *Rhodococcus aetherivorans*, *phaJ_pa_* from *Pseudomonas aeruginosa*, and *phaA_Re_* from *Ralstonia eutropha*		[22]
pPhaCJ	pBBR1MCS-2-based plasmid with *Ralstonia eutropha*-optimized sequence of *phaC_Ra_* and *phaJ_Pa_*		This study

**Table 2:** Analysis of EPS composition using GC-MS.

EPS Composition	Amount (%, Peak Area)
β-D-glucopyranose	49.66 ± 2.73
β-D-Talopyranose	35.55 ± 1.76
L-Rhamnose	10.14 ± 0.82
L-Rhamnopyranose	4.63 ± 0.14

**Table 3:** Analysis of physical properties via GPC.

	Mn	Mw	PDI
BM3-1 (PHB)	8.85 × 10^5^	1.19 × 10^6^	1.34
BM3-1 pCB81 (5.9 mol% 3HHx)	3.67 × 10^5^	5.88 × 10^5^	1.60
BM3-1 pPhaCJ (3.5 mol% 3HHx)	3.65 × 10^5^	5.64 × 10^5^	1.54

**Table 4:** Analysis of physical properties via UTM.

	Tensile Strength (Mpa)	Elongation at Break (%)	Young’s Modulus (Mpa)
BM3-1 (PHB)	7.83	24.8	407.8
BM3-1 pCB81 (5.9 mol% 3HHx)	5.22	99.1	86.2
BM3-1 pPhaCJ (3.5 mol% 3HHx)	6.54	67.8	137.5

**Table 5:** Analysis of thermal properties via DSC. (N.D. stands for non-detection).

	Tg (°C)	Tm (°C)	Tc (°C)
BM3-1 (PHB)	N.D.	174.3	96.0
BM3-1 pCB81 (5.9 mol% 3HHx)	−2.4	163.2	N.D.
BM3-1 pPhaCJ (3.5 mol% 3HHx)	−3.3	170.2	47.1

## Data Availability

Data are contained within the article.
